# Editorial: Immunological and virological aspects of the pathogenesis of type 1 diabetes

**DOI:** 10.3389/fimmu.2025.1688257

**Published:** 2025-11-06

**Authors:** Didier Hober

**Affiliations:** Laboratoire de Virologie ULR3610, Univ Lille, Centre Hospitalier Universitaire de Lille, Lille, France

**Keywords:** type 1 diabetes, immunology, virology, pathogenisis, mechanisms

Research is underway to better understand the pathogenesis of type 1 diabetes mellitus (T1DM). A better understanding of the pathogenesis should open avenues for developing new approaches to patient management and might lead to the development of prevention strategies. Viruses of the *Enterovirus* genus, in particular coxsackievirus B (CVB), could play a role in the pathogenesis of T1DM. A Research Topic in *Frontiers in Immunology* is dedicated to the immunological and virological aspects of the pathogenesis of T1DM.

Lemos et al. in their paper entitled *Immunological and virological triggers of type 1 diabetes: insights and implications* examine the intricate relationship between viral infections and autoimmunity and discuss potential considerations for prevention and treatment strategies (DOI 10.3389/fimmu.2023.1326711). How can viral infections accelerate the development of T1DM? The mechanisms linking viral infections to beta-cell death are presented: direct infection of islets by viruses, indirect fashion by modulating the immune system, and stress on the beta-cell. Viral infections can accelerate the autoimmune process leading to T1DM. In addition to enteroviruses, the authors describe the possible role of viruses such as SARS-CoV-2, herpesviruses, and rotavirus.

Lalani et al. used immortalized trophoblast cells infected with CVB4 to identify microRNAs. A novel microRNA has the potential to be an early biomarker of CVB4-induced type 1 diabetes. The inhibition of this microRNA can reduce the replication of the virus, suggesting that targeting microRNAs may be a relevant approach to the fight against CVB-induced T1DM.

A non-synonymous single-nucleotide polymorphism (SNP) associated with T1D is in the interferon-induced helicase C domain-containing protein 1 (IFIH1), which encodes an anti-viral cytosolic RNA sensor. Substitution of one amino acid (IFIH1A946T) confers an increased risk for T1D. In IFIH1A946T risk variant (IFIH1^R^) knock-in mice from a non-obese diabetic (NOD) mouse background, Stock et al. observed a significant acceleration of diabetes onset in (IFIH1^R^) females compared to non-risk (IFIH1^NR^) mice. In (IFIH1^R^) mice the frequency and activation of immune cells were altered. The data indicate that IFIH1^R^ may contribute to T1D pathogenesis.

T1DM has a significant impact on the whole life of diabetic patients due to related complications, especially macrovascular complications. There is a heterogeneity in T1DM and, therefore, the risk of complications varies markedly between patients. In the paper entitled *Blood immune cell profiling in adults with longstanding type 1 diabetes is associated with macrovascular complications*, He et al. studied the blood immune cell profile of adult patients with longstanding T1DM and healthy controls by FACS analysis, followed by a machine-learning based elastic-net classification model. This study shows that there are two distinct immunological profiles in adults with longstanding type 1 diabetes; one group of patients has a stronger pro-inflammatory profile and is associated with a higher rate of diabetes-related macrovascular complications.

The survival and function of islet allografts and the prevention of T1DM are two major concerns. These issues have been addressed in murine models of transplantation and in NOD mice by Chuang et al. This team developed small-molecule inhibitors (SMIs) of the CD40- CD40L(CD154) costimulatory protein-protein interaction and, in this paper, analyzed the effect of two such SMIS: DRI-C21041 and DRI-C21095. This study shows that SMIs can inhibit the TNF superfamily protein-protein interaction and that these molecules through CD40-CD40L blockade can be useful in islet transplantation and T1D prevention.

T1DM can be an adverse reaction to immunotherapy. A patient developed T1DM following treatment with Envafolimab, a PD-L1 immune checkpoint inhibitor. T1DM was induced by the immunomodulating effects of Envafolimab and not by a pre-existing autoimmune condition because diabetes-related autoantibodies were absent in this patient. This case report draws attention to the fact that studies are needed to elucidate the mechanisms of the diabetogenic effect of immunotherapy to better understand the pathogenesis of T1DM (Li et al.).

The Research Topic in *Frontiers in Immunology* dedicated to the immunological and virological aspects of the pathogenesis of T1DM highlights that research is underway to better understand the pathogenesis of this autoimmune disease and to develop strategies to improve the management of patients.

